# Impact of Mobilization Strategies on Peripheral Blood Stem Cell Collection Efficiency and Product Quality: A Retrospective Single-Center Study

**DOI:** 10.3390/cancers14246259

**Published:** 2022-12-19

**Authors:** Patricija Rajsp, Manuela Branka, Nelly Besson, Andreas Tanzmann, Nina Worel

**Affiliations:** 1 Department of Blood Group Serology and Transfusion Medicine, Medical University of Vienna, Waehringer Guertel 18-20, A-1090 Vienna, Austria; 2 Medical Affairs Department, Terumo Blood and Cell Technologies, Ikaroslaan 41, 1930 Zaventem, Belgium

**Keywords:** peripheral blood stem cell collection, apheresis, autologous peripheral blood stem cell transplantation, Spectra Optia^®^ continuous mononuclear cell collection protocol, plerixafor

## Abstract

**Simple Summary:**

We retrospectively reviewed data from cancer patients undergoing peripheral blood stem cell (PBSC) collection following different mobilization regimens (with or without plerixafor) for autologous stem cell transplantation. We used the Spectra Optia^®^ continuous mononuclear platform for stem cell collections. The collection efficiency of CD34+ cells and other product characteristics of 210 PBSC collections following mobilization without plerixafor and 99 collections with plerixafor were assessed. We observed similar product characteristics between the two mobilization regimens. Mobilization with plerixafor slightly increased CD34+ collection efficacy, but the difference was negligible. We showed that the Spectra Optia^®^ apheresis system leads to similar CD34+ cell collection efficacy and good-quality PBSC products for mobilization regimens with and without plerixafor.

**Abstract:**

Autologous stem cell transplantation is routinely used in the management of several hematological diseases, solid tumors, and immune disorders. Peripheral blood stem cell (PBSC) collection performed by apheresis is the preferred source of stem cells. In this study, the potential impact of mobilization regimens on the performance of the Spectra Optia^®^ continuous mononuclear cell collection system was evaluated. We performed a retrospective data analysis for patients undergoing autologous PBSC collection at the Medical University Vienna, Vienna General Hospital between September 2016 and June 2018. Collections were divided into two main groups according to the mobilization regimen received: without (210 collections) or with (99 collections) plerixafor. Assessed variables included product characteristics and collection efficiency (CE). Overall, product characteristics were similar between the groups. Median CD34+ CE2 was 50.1% versus 53.0%, and CE1 was 66.9% versus 69.9% following mobilization without and with plerixafor, respectively; the difference was not statistically significant. Simple linear regression showed a very weak positive correlation between the mobilization method and CE1 or CE2 (mobilization with plerixafor increased CE2 by 4.106%). In conclusion, the Spectra Optia^®^ apheresis system led to high CE and a good quality of PBSC products when mobilization regimens with or without plerixafor were used.

## 1. Introduction

Autologous stem cell transplantation in combination with high-dose chemotherapy (CT) has become the standard of care for several hematological diseases and immune disorders [1]. A significant improvement in progression-free survival was shown, for example, for patients with multiple myeloma and Hodgkin’s and non-Hodgkin’s lymphomas after high-dose CT/autologous stem cell transplantation [2,3,4,5]. Furthermore, this approach has already become a recognized therapy option for several solid tumors, such as germ cell tumors, osteosarcoma, or neuroblastoma [5,6,7].

For more than three decades, peripheral blood stem cell (PBSC) collection performed by apheresis has been the preferred source of stem cells, as it has several advantages compared with bone marrow collection. The use of PBSC has been associated with faster recovery of neutrophils and platelets and a higher overall probability of overall and disease-free survival than bone marrow transplantation [8]. In PBSC collection, the egress of stem cells in peripheral blood is stimulated with mobilizing regimens that standardly include granulocyte colony-stimulating factor (G-CSF) with or without CT and the use of a chemokine receptor 4 (CXCR4) antagonist such as plerixafor for poor mobilizers [9]. A minimum of 2 × 10^6^ CD34+ cells/kg recipient body weight (b.w.) is required for successful engraftment. However, a dose-dependent response was observed, and higher doses of up to 5 × 10^6^ CD34+ cells/kg induce better neutrophil and platelet recovery [10,11,12,13]. Sufficient mobilization of hematopoietic progenitor cells in the peripheral blood depends on various factors, including age, sex, diagnosis, bone marrow involvement, pretreatment including irradiation, and mobilization regimen used [13].

A major challenge in PBSC collection remains in obtaining the necessary amount of hematopoietic progenitor cells while planning the duration of the procedure to be as short as possible to avoid patient discomfort (e.g., reactions to the anticoagulation solution and positional pain) and not to strain staff resources. Various algorithms for yield prediction in leukapheresis have been developed in the past, but without resounding success [14,15,16,17,18,19,20,21]. In addition to the CD34+ cell count in the peripheral blood prior to collection and the volume of processed blood [22,23,24], one of the variables impacting the result of apheresis is the collection efficiency (CE) of the device used. Significant fluctuations, which cannot be fully explained yet, sometimes occur. In addition, other factors can influence device efficiency, such as CD34+ cell recruitment and kinetics during apheresis [25,26]. Intra-apheresis CD34+ cell recruitment has not been fully investigated but could also be influenced by the mobilization regimens, which sometimes vary even for the same diagnosis.

It is recommended that prediction algorithms for optimizing collection yields are developed and implemented separately by each center. This allows for consideration of the patient population, transplantation goals, employed apheresis technique and device, cost limitations, and mobilization strategies, which may differ substantially between centers [24]. We have therefore compared the impact of mobilization regimens on PBSC collection in our center using the same apheresis system to assess its performance in terms of collection yield and product characteristics.

## 2. Study Design and Methods

### 2.1. Cohorts and Data Collection

We retrospectively reviewed data from patients undergoing autologous PBSC collection at the Medical University of Vienna, Vienna General Hospital between September 2016 and June 2018. Patients were divided into two main groups depending on which mobilization regimen they received, i.e., with or without plerixafor, as decided by the transfusion medicine specialists. Mobilizations were further stratified by use of chemotherapeutic agents into four subgroups: steady state with G-CSF alone (G-CSF), G-CSF and CT (G-CSF + CT), steady state with G-CSF and plerixafor (G-CSF + plerixafor), and CT with G-CSF and plerixafor (G-CSF + CT + plerixafor). The choice between G-CSF and G-CSF + CT mobilization was made by the referring physicians, based on their professional experience and assessment of the patients’ medical history in accordance with the standard protocols of our institution. In a few cases, a second mobilization cycle was necessary to achieve the required transplant dose; subsequent collection was only included in the analysis if the mobilization regimen differed from the first one. All consecutive collections with complete data were included in this study.

Collected data included baseline demographic characteristics (patient age, sex, diagnosis, total blood volume (TBV), and b.w.), procedural characteristics (procedure time, whole blood volume processed), pre- and post-apheresis peripheral blood CD34+ cell counts, and other hematological parameters (pre- and post-apheresis white blood cell, platelet, hematocrit, and granulocyte counts), and product characteristics.

The study was conducted according to the Good Clinical Practice guidelines and was approved by the Ethics Committee of the Medical University of Vienna (EC 1362/2022).

### 2.2. Stem Cell Mobilization

The target dose of CD34+ cells varied from 3 × 10^6^ to 9 × 10^6^ CD34+ cells/kg b.w. depending on the request of the referring physicians (i.e., one, two, or three required autologous stem cell transplantations).

Patients were injected subcutaneously with 5 μg/kg b.w. of G-CSF (filgrastim; e.g., *Neupogen* (Amgen, Thousand Oaks, CA, USA), *Zarzio* (Sandoz, Basel, Switzerland), or other biosimilars)) twice daily for at least four days before collection. In patients receiving CT, G-CSF stimulation was started 5 to 7 days after CT initiation. Leukapheresis was started when CD34+ levels reached values of ≥20 cells/µL and nucleated cell count reached 5 × 10^9^/L.

Plerixafor (*Mozobil* (Genzyme, Cambridge, MA, USA)) was administered when CD34+ counts had not increased sufficiently (i.e., not increased to ≥20 CD34+ cells/µL) on the expected collection day despite a white blood cell count of approximately 10,000/µL and there were no contraindications according to the product information (e.g., acute leukemia). Patients received plerixafor at 0.24 mg/kg b.w. or 0.16 mg/kg b.w. (in the case of renal insufficiency and creatinine clearance <50 mL/min) approximately 8 to 11 h before the start of the PBSC collection [27,28,29].

### 2.3. Blood Count Parameters

Cell counts from pre-collection peripheral blood and from the final product were generated by a hematology analyzer (Beckmann Coulter DxH 600). CD34+ cells were counted according to the International Society for Hematotherapy and Graft Engineering (ISHAGE) protocol using a flow cytometer (Becton Dickinson FACSCanto™ II system). Apheresis was initiated only if the hemoglobin level was >8 g/dL and the platelet count >20 × 10^9^/L. A minimum leukocyte number of 5 × 10^9^/L and 20 CD34+ cells/µL was required to avoid the risk of collection failure. However, PBSC collections were also initiated with lower CD34+ cell counts in the case of poor mobilizers after administration of plerixafor. Patients with hemoglobin levels of <8 g/dL or platelet counts of <15 × 20 × 10^9^/L received red cell or platelet support, respectively.

### 2.4. Apheresis

PBSC collection was performed with the Spectra Optia^®^ device (Terumo Blood and Cell Technologies, Lakewood, CO, USA) with the continuous mononuclear cell collection (cMNC) protocol (software version 11).

Venous access was achieved either by peripheral or central veins. The anticoagulant used was acid–citrate–dextrose formula A (ACD-A) at a standard ratio of 1:12 or ACD-A with heparin (4500 IU per 750 mL ACD-A) at a ratio of 1:22 when more than three times the patient’s TBV was processed. 

### 2.5. Procedure Performance

Procedure performance was evaluated based on CD34+ cell CE, recruitment factor, change, and throughput (TP) coefficients, using Equations (1)–(6):

(1) $$\mathrm{CD}34+\mathrm{CE}2\;(\%)=\frac{{\mathrm{Total}\;\mathrm{CD}34+\mathrm{cells}\;\mathrm{in}\;\mathrm{the}\;\mathrm{product}\;(\times 10}^{6})}{{\mathrm{Pre}-\mathrm{CD}34+\mathrm{in}\;\mathrm{peripheral}\;\mathrm{blood}\;(\times 10}^{6}/L)*\mathrm{Blood}\;\mathrm{processed}\;\mathrm{without}\;\mathrm{anticoagulant}\;(L)}*100$$

(2) $$\mathrm{CD}34+\mathrm{CE}1(\%)=\frac{{\mathrm{Total}\;\mathrm{CD}34+\mathrm{cells}\;\mathrm{in}\;\mathrm{the}\;\mathrm{product}\;(\times 10}^{6})}{\frac{\mathrm{Pre}-\mathrm{CD}34++\mathrm{Post}-\mathrm{CD}34+}{2}{\;(\mathrm{in}\;\mathrm{peripheral}\;\mathrm{blood}\;,\;\times 10}^{6}/L)*\mathrm{Blood}\;\mathrm{processed}\;\mathrm{without}\;\mathrm{anticoagulant}\;(L)}*100$$

(3) $$\mathrm{CD}34+\mathrm{recruitment}\;\mathrm{factor}=\frac{{\mathrm{Total}\;\mathrm{CD}34+\mathrm{cells}\;\mathrm{in}\;\mathrm{the}\;\mathrm{product}\;(\times 10}^{6})+(\mathrm{Post}-\mathrm{CD}34+\mathrm{in}\;\mathrm{peripheral}\;\mathrm{blood}\;\left(\times \frac{10^{6}}{L}\right)*\mathrm{TBV}\;\left(L\right))}{{\mathrm{Pre}-\mathrm{CD}34+\mathrm{in}\;\mathrm{peripheral}\;\mathrm{blood}\;(\times 10}^{6}/L)*\mathrm{TBV}\;(L)}$$

(4) $$\mathrm{CD}34+\mathrm{change}=1-\frac{{\mathrm{Post}-\mathrm{CD}34+\mathrm{in}\;\mathrm{peripheral}\;\mathrm{blood}\;(\times 10}^{6}/L)}{{\mathrm{Pre}-\mathrm{CD}34+\mathrm{in}\;\mathrm{peripheral}\;\mathrm{blood}\;(\times 10}^{6}/L)}$$

(5) $$\mathrm{CD}34+\mathrm{TP}2=\frac{{\mathrm{Total}\;\mathrm{CD}34+\mathrm{cells}\;\mathrm{in}\;\mathrm{the}\;\mathrm{product}\;(\times 10}^{6})}{{\mathrm{Pre}-\mathrm{CD}34+\mathrm{in}\;\mathrm{peripheral}\;\mathrm{blood}\;(\times 10}^{6}/L)*\mathrm{Procedure}\;\mathrm{time}}$$

(6) $$\mathrm{CD}34+\mathrm{TP}1=\frac{{\mathrm{Total}\;\mathrm{CD}34+\mathrm{cells}\;\mathrm{in}\;\mathrm{the}\;\mathrm{product}\;(\times 10}^{6})}{\;\frac{\mathrm{Pre}-\mathrm{CD}34++\mathrm{Post}-\mathrm{CD}34+}{2}\;\left({\mathrm{in}\;\mathrm{peripheral}\;\mathrm{blood}\;\times 10}^{6}/L\right)*\mathrm{Procedure}\;\mathrm{time}}$$

Platelet loss in percent (%) was calculated as follows:

(7) $$\mathrm{Platelet}\;\mathrm{loss}(\%)=\frac{{\mathrm{Post}-\mathrm{count}\;(\times 10}^{9}/L)}{{\mathrm{Pre}-\mathrm{count}\;(\times 10}^{9}/L)}*100$$

CE1 for a specific cell population is based on the average of pre- and post-apheresis counts in the peripheral blood. CE2 is based on the principle that CD34+ cell collection is reasonably uniform over the course of a PBSC collection (i.e., a dynamic equilibrium is maintained in which CD34+ cells are recruited to the peripheral blood to replace those removed by apheresis). CE2 is therefore a calculated value from the variables of pre-apheresis peripheral blood cell count and the amount of blood volume processed in total. The result is given as a percentage of cells collected from the amount of cells in the collection bag at the end of apheresis [15].

### 2.6. Statistical Analysis

Patient characteristics and procedural performance data were presented using descriptive statistics (median values and minimum–maximum range). Differences between the two main groups (without or with plerixafor) were analyzed using the nonparametric Mann–Whitney U test, with the statistical significance limit set at 0.05. For further comparison between the four subgroups (G-CSF alone, G-CSF + plerixafor, G-CSF + CT, and G-CSP + CT + plerixafor), the Kruskal–Wallis test was used.

The correlation between pre-harvest CD34+ cells and CD34+ cells collected per liter of blood processed was analyzed using linear regression, with values for R^2^ > 0.8 indicating good prediction.

The impact of the mobilization regimen (without or with plerixafor) on CE (CD34+ CE1 and CE2) was assessed using simple linear regression with ANOVA.

Analyses were performed using SPSS v27.

## 3. Results

We reviewed 369 collections. Data from 309 collections were completely available and were included in the analyses; for 210 (68.0%) and 99 (32.0%) collections, mobilization was performed without and with plerixafor, respectively (Table 1). The group without plerixafor included 97 (31.4%) collections with G-CSF alone and 113 (36.6%) with G-CSF + CT, whereas the plerixafor group consisted of 64 (20.7%) collections with G-CSF + plerixafor, and 35 (11.3%) collections with G-CSF + CT + plerixafor. Each patient underwent 1–3 collections, with multiple collections performed within 2 to 3 consecutive days. For the majority of patients, only one mobilization cycle was necessary.

Patient characteristics and pre-apheresis blood counts are summarized in Table 1. Slightly more than half of all procedures (177; 57.3%) were performed in patients diagnosed with multiple myeloma, although this proportion differed between mobilization regimens (53.3% for collections without plerixafor versus 65.7% for collections with plerixafor). Patients with multiple myeloma were most often mobilized with G-CSF alone or with G-CSF and plerixafor. The majority of patients underwent apheresis with peripheral venous access; for nearly all patients for whom peripheral venous access was possible, the apheresis was performed in the outpatient setting (data not shown).

Patients in the plerixafor group were significantly older and had significantly lower pre-apheresis platelet counts than those in the group without plerixafor; both higher age and low pre-apheresis platelet counts are known risk factors for poor mobilization. Pre-apheresis CD34+ counts indicated adequate mobilization in most patients, with a statistically significant higher median cell count observed for patients not requiring plerixafor (Table 1). As a consequence, the median post-apheresis peripheral CD34+ cell count was significantly higher for mobilizations without plerixafor (22.1 (range 1.0–588.0)) compared to mobilization with plerixafor (12.5 (range 1.5–73.6)) (*p* < 0.001; Table 2). No correlation was found between CE1 or CE2 and pre-apheresis CD34+ counts (Figure S1).

Post-apheresis median white blood cell and granulocyte counts were higher while platelet count, total CD34+ cell count in the collection bag (10^6^), and CD34+ yield (10^6^/kg b.w.) were lower for collections following mobilization with plerixafor than without plerixafor (Table 2).

**Figure 1:**
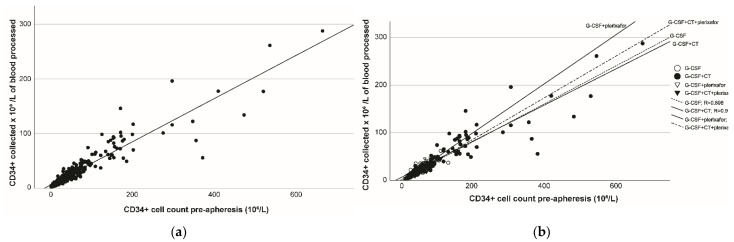
Correlations between pre-apheresis CD34+ cell count and CD34+ cells collected per liter of blood processed (**a**) overall and (**b**) by mobilization regimen. G-CSF, granulocyte colony-stimulating factor; CT, chemotherapy.

A strong linear correlation was observed between the CD34+ pre-apheresis count and CD34+ cells collected per liter of blood processed, with R^2^ = 0.866 (R = 0.930; Figure 1a). R^2^ values higher than 0.8 were still observed for each mobilization regimen (Figure 1b), indicating that this correlation is not impacted by the mobilization regimen.

**Figure 2:**
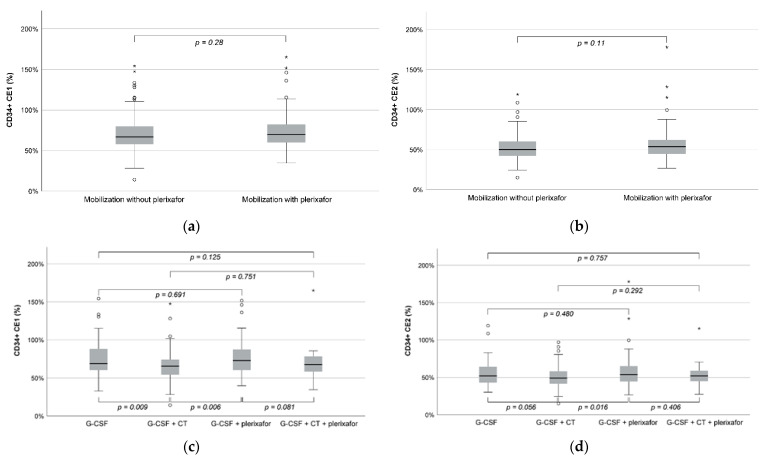
Distribution of individual CE1 (**a**,**c**) and CE2 (**b**,**d**) values by mobilization regimen (**a**,**b**) and subgroup (**c**,**d**). CE, collection efficiency; Q1, first quartile; Q3, third quartile; G-CSF, granulocyte colony-stimulating factor; CT, chemotherapy. Notes: Error bars represent 95% confidence intervals. Outliers (◦) are defined as values lower than Q1-1.5*interquartile range or higher than Q3 + 1.5*interquartile range. Extreme outliers (*) are defined as values lower than Q1-3*interquartile range or higher than Q3 + 3*interquartile range.

Higher median whole blood volume (19.4 L versus 17.7 L; *p* = 0.004) and TBV (4.0 versus 3.9 times; *p* < 0.001) processed and longer duration of apheresis (333 versus 314 min; *p* = 0.002) were noted for mobilization with plerixafor. However, no statistically significant difference was observed between mobilization without or with plerixafor for CD34+ CE2 (50.1% versus 53.0%; *p* = 0.11) and CD34+ CE1 (66.9% versus 69.9%; *p* = 0.28) (Table 2, Figure 2a,b). The median CD34+ recruitment factor was lower for collections without than with plerixafor (2.3 versus 2.7; *p* < 0.001) (Table 2).

When further stratified by mobilization regimen with or without CT, mobilization with G-CSF only afforded the product with the highest platelet contamination but also the highest platelet loss in the patient, with statistically significant differences compared with the other subgroups (Table 3 and Table S1). Mobilization with G-CSF + CT yielded the product with the highest count of CD34+ cells collected per kg b.w. However, CD34+ CE2, TP1, and TP2 were similar between the four subgroups, with the only statistically significant difference being observed for a lower CD34+ CE2 in the G-CSF + CT subgroup versus the G-CSF + plerixafor subgroup and a lower CD34+ CE1 in the G-CSF + CT subgroup versus the G-CSF and the G-CSF + plerixafor subgroups (Table 3 and Table S1).

Simple linear regression showed a very weak positive correlation between the two groups (mobilization without or with plerixafor) and CE1 or CE2 (Figure S1); however, a statistically significant impact was shown with ANOVA only for CE2. The regression function indicated that mobilization with plerixafor increases CE2 by 4.106% compared with the regimen without plerixafor (Equation (8); Table S2).

(8) y=52.205+4.106 x

Pre-apheresis platelet levels varied depending on the mobilization regimen used (Table 1). Values in the plerixafor group were statistically significantly lower than in the group without plerixafor, with platelet counts of <30 × 10^9^/L for 18 patients. However, the post-apheresis platelet counts did not decrease below 10 × 10^9^/L in any of these 18 patients and none experienced bleeding complications. Platelet loss (Equation (7)) directly correlated with pre-collection platelet counts in the peripheral blood (Figure S2). Correspondingly, the harvested products showed a higher platelet contamination in patients with higher pre-collection counts. Median platelet loss was 35.2% for mobilizations without plerixafor and 34.6% for mobilizations with plerixafor; no statistically significant difference was observed between the two groups (*p* = 0.491; Table 2). When stratified in subgroups, higher loss was observed for G-CSF mobilizations without plerixafor (42.2%) compared with the G-CSF group with plerixafor (36.5%), with statistically significant differences observed between all subgroups except the G-CSF + CT versus the G-CSF + CT + plerixafor group (Table 3 and Table S1, Figure 2c,d).

## 4. Discussion

The efficacy and safety of PBSC collection using the Spectra Optia^®^ cMNC protocol has previously been shown in both mobilized and non-mobilized patients or healthy donors with a wide range of diagnoses [30,31,32,33,34]. Efficiency is improved compared with the non-continuous protocol on the same system even in patients with a low pre-apheresis CD34+ cell count [30]. Our study supports previous observations and further shows a similar performance of the Spectra Optia^®^ cMNC protocol, regardless of the mobilization regimens in terms of CD34+ CE1, CD34+ CE2, and other product characteristics. We compared mobilization regimens with or without plerixafor and then further stratified these results by the use of CT-based mobilization. As a result, we found that CD34+ CE1 and CE2 were nearly similar; a regression analysis showed a weak significantly higher CE2 efficiency when plerixafor was used, which is clinically negligible. This similarity between mobilizations regardless of the use of plerixafor for stem cell egress in the peripheral blood was noted despite significantly lower pre-apheresis CD34+ cell counts in poor mobilizers requiring plerixafor, for whom poorer yields are expected.

The product characteristics were overall similar between groups receiving plerixafor or not, and median values for white blood cell and platelet counts and hematocrit were in line with previous reports for the same apheresis system [31,32,35,36]. Platelet loss has been shown to be reduced for the MNC platform of the Spectra Optia^®^ system compared with other apheresis systems [35,37] and is also lower compared with the cMNC protocol [30]. In this study, we found a difference in the median values for platelet loss following apheresis between patients without and with plerixafor mobilizations with the highest loss in G-CSF alone, followed by G-CSF + plerixafor, G-CSF + CT + plerixafor, and G-CSF + CT. The value for the G-CSF group was also higher than the decrease observed in a study in 32 patients with multiple myeloma receiving the same mobilization regimen and for whom PBSC collection was performed with the same apheresis system but with the MNC protocol (38.1%) [35]. Furthermore, we observed a higher median value for platelet CE1 in the G-CSF group in this study and no statistically significant difference for platelet loss between patients mobilized without or with plerixafor. Although a higher platelet loss in patients with higher platelet counts was observed, no bleeding complications occurred. Of note, pre-apheresis platelet counts were significantly lower in the group with plerixafor than in the group without plerixafor, indicating that one risk factor for poor mobilizers is a low platelet count at mobilization [24,38]. In addition, CT-based mobilization is known to result in temporary myelosuppression including thrombocytopenia. For a safe apheresis procedure, patients should have platelet counts of >20–30 × 10^9^/L. Our study included 18 patients with pre-apheresis platelet levels of <30 × 10^9^/L, but none of them experienced a decrease in platelets during stem cell harvest to levels of <10 × 10^9^/L, and therefore there was no risk of spontaneous bleeding.

In our study, median CD34+ cell counts (total and per kg b.w.) for the final product were higher following mobilization without than with plerixafor, and significant differences in pre-apheresis CD34+ cell counts were also observed. As in previous studies [14,17,18], we found a strong correlation between pre-apheresis CD34+ cell count and CD34+ cell dose collected, indicating that the CD34+ cell final dose can be predicted with sufficient accuracy and that pre-apheresis CD34+ cell count is the strongest predictor of PBSC collection outcome.

Mobilization with plerixafor led to only slightly higher volumes of whole blood processed, TBV per runs, and apheresis duration compared with collections without plerixafor. In this study, in accordance with the protocol at our site, the processing of larger blood volumes in a single procedure was preferred. Processing of large blood volumes have been previously shown to improve CD34+ cell yield even in poor mobilizers, which can lead to a reduction in the cost of the procedure (i.e., lower number of aphereses) and risk of adverse events [14,39,40].

Overall, performance characteristics observed in our study were in line with those in other studies in the adult population with the same apheresis device [30,31,35,36,41,42,43,44,45,46]. We found similar median CE1 and CE2 for mobilization without or with plerixafor, showing that an efficient collection can be achieved regardless of the initial mobilization status (adequate or poor). A statistically significant higher median CD34+ recruitment factor was observed following mobilization with plerixafor than without, further indicating efficient recruitment of CD34+ cells in the peripheral blood with plerixafor. Plerixafor is widely accepted as an effective and safe mobilization strategy in combination with either G-CSF alone or G-CSF and CT in poor mobilizers [23,27,47], while also being cost-efficient [48,49]. Median CE1 and CE2 values varied from 65.6% to 72.9% and from 49.1% to 54.0%, respectively, across the four subgroups evaluated in this study. CE1 can be viewed as a more accurate parameter as it involves both pre- and post-apheresis CD34+ cell counts. A statistically significant difference was only observed for median CD34+ CE1 in subgroups receiving either G-CSF or G-CSF + plerixafor versus those mobilized with G-CSF + CT, with slightly higher values noted in the same group (without or with plerixafor) when CT-based mobilization was not used.

The main strength of this study is that it allowed the comparison of different mobilization strategies using the same apheresis device and protocol, thus limiting the consequences of device variability on the evaluation of mobilization regimens. These data will serve as a basis for a future study in which we aim to validate performance prediction algorithms for autologous PBSC collection. However, the current study also has several limitations. First, the retrospective rather than prospective design of the study led to inherent limitations. However, data were collected consistently, limiting potential bias. There were also differences in patient characteristics between groups, although these reflect real-life situations. For some analyses, the sample size was relatively low, thus hindering the interpretation of comparisons between subgroups.

## 5. Conclusions

The use of the Spectra Optia^®^ apheresis system with the cMNC protocol led to high CE1 and CE2 and good-quality PBSC products. Our results confirm previous observations on the performance of Spectra Optia^®^ systems in PBSC collection when using mobilization regimens with and without plerixafor.

## Figures and Tables

**Table 1:** Patient characteristics and pre-apheresis peripheral blood counts for the reviewed peripheral blood stem cell collection procedures.

Variables	Mobilization without Plerixafor	Mobilization with Plerixafor	*p*-Value
Number of collections, n (%) *	210 (68.0%)	99 (32.0%)	
G-CSF	97 (31.4% **)	64 (20.7% **)	0.02
G-CSF + CT	113 (36.6% **)	35 (11.3% **)	0.02
Gender (male), n (%)	143 (68.1%)	68 (68.7%)	0.917
G-CSF	67 (69.1% ***)	48 (75.0% ***)	
G-CSF + CT	76 (67.3% ***)	20 (57.1% ***)	
Age (years), median (range)	54 (19–74)	58 (23–76)	0.016
G-CSF	56 (19–74)	61 (24–75)	
G-CSF + CT	51 (20–73)	52 (23–76)	
Weight (kg), median (range)	80 (42–150)	82 (48–120)	0.588
G-CSF	83 (52–124)	82 (54–117)	
G-CSF + CT	76 (42–150)	79 (48–120)	
TBV (L), median (range)	5.1 (2.7–7.4)	5.1 (3.1–6.9)	0.689
G-CSF	5.1 (3.3–6.9)	5.2 (3.4–6.5)	
G-CSF + CT	5.0 (2.7–7.4)	4.6 (3.1–6.9)	
Diagnosis, n (%)			
Multiple myeloma	112 (53.3%)	65 (65.7%)	
Non-Hodgkin’s lymphoma	69 (32.9%)	21 (21.2%)	
Hodgkin’s disease	6 (2.9%)	7 (7.1%)	
Other carcinoma	22 (10.5%)	6 (6.1%)	
Non-malignant disease	1 (0.5%)	0 (0.0%)	
Pre-apheresis peripheral blood counts			
White blood cells (10^9^/L)	36.1 (6.5–132.0)	42.8 (6.2–77.1)	0.001
G-CSF	44.8 (17.1–88.0)	44.5 (21.6–77.1)	
G-CSF + CT	25.0 (6.5–132.0)	37.2 (6.2–74.7)	
Platelets (10^9^/L)	134 (15–562)	104 (20–296)	0.042
G-CSF	198 (57–562)	136 (52–296)	
G-CSF + CT	80 (15–304)	64.0 (20–246)	
Hematocrit (%)	34.8 (20.4–49.9)	33.2 (22.1–45.1)	0.206
G-CSF	38.0 (24.0–50.0)	36.8 (25.1–45.1)	
G-CSF + CT	31.2 (20.4–44.3)	29.7 (22.1–42.2)	
Granulocytes (%)	85 (50–95)	84 (58–95)	0.372
G-CSF	86 (71–93)	83 (69–95)	
G-CSF + CT	84 (50–95)	87 (58–95)	
CD34+ cells (×10^6^/L)	47.4 (3.8–663.0)	28.2 (2.5–80.8)	<0.001
G-CSF	33.2 (3.8–162.8)	30.6 (2.5–80.8)	
G-CSF + CT	75.4 (8.6–663.0)	20.3 (4.7–69.6)	
CD34+ cells (%)	0.1 (0.0–2.7)	0.1 (0.0–0.3)	<0.001
G-CSF	0.1 (0.0–0.3)	0.1 (0.0–0.2)	
G-CSF + CT	0.3 (0.0–2.7)	0.1 (0.0–0.3)	

n (%), number (%) of collections in each category; G-CSF, granulocyte colony-stimulating factor; CT, chemotherapy; TBV, total blood volume. Note: * the number of patients in each subgroup was 80 (G-CSF), 55 (G-CSF + plerixafor), 106 (G-CSF + CT), 30 (G-CSF + CT + plerixafor); ** percentages calculated using the number of collections in each of the main groups (mobilization with or without plerixafor) as denominator; *** percentages calculated using the number of collections in each of the four subgroups (G-CSF, G-CSF + CT, G-CSF + plerixafor, G-CSF + CT + plerixafor) as denominator. Other data are presented as median values with minimum–maximum ranges.

**Table 2:** Product characteristics and performance specifications for the Spectra Optia^®^ continuous mononuclear cell collection protocol for mobilization regimens without or with plerixafor.

Variables	Mobilization Without Plerixafor	Mobilization with Plerixafor	*p*-Value
Whole blood processed (L)	17.7 (6.4–29.4)	19.4 (10.7–28.9)	0.004
TBV processed (× times)	3.9 (1.4–5.7)	4.0 (2.0–5.3)	<0.001
White blood cells (×10^9^/L)	194.0 (44.0–633.0)	214.0 (48.1–569.0)	0.021
Platelets (×10^9^/L)	1015 (29–4980)	765 (71–4505)	0.034
Hematocrit (%)	1.4 (0.0–5.3)	1.1 (0.0–4.2)	0.069
Granulocytes (%)	7 (0–67)	13 (0–71)	0.010
CD34+ cells total in bag (10^6^)	400 (20–2800)	270 (30–1200)	0.001
CD34+ cells collected (10^6^/kg b.w.)	5.7 (0.2–34.9)	3.1 (0.5–11.3)	<0.001
Post-apheresis CD34+ cells (×10^6^/L)	22.1 (1.0–588.0)	12.5 (1.5–73.6)	<0.001
G-CSF	14.1 (2.0–103.0)	11.7 (1.7–73.6)	
G-CSF + CT	41.1 (1.0–588.0)	12.5 (1.5–63.0)	
CD34+ change (%)	51.1 (−153.7–96.5)	51.0 (−127.4–90.7)	0.384
G-CSF	57.4 (−153.7–89.7)	53.0 (−127.4–90.7)	
G-CSF + CT	48.6 (−86.5–96.5)	41.4 (−10.1–73.5)	
Apheresis duration (minutes)	314 (140–510)	333 (186–480)	0.002
CD34+ CE2 (%)	50.1 (15.0–119.1)	53.0 (26.7–178.0)	0.11
CD34+ CE1 (%)	66.9 (14.2–154.4)	69.9 (34.6–165.1)	0.28
CD34+ TP2	3.1 (0.8–7.1)	3.3 (1.5–9.5)	0.084
CD34+ TP1	3.9 (0.8–9.2)	4.1 (1.8–10.2)	0.291
CD34+ recruitment factor	2.3 (1.1–5.5)	2.7 (1.3–9.3)	<0.001
Platelet loss (%)	35.2 (0.0–60.8)	34.6 (3.9–61.3)	0.491
Platelet CE1 (%)	13.9 (2.9–57.6)	14.1 (5.6–70.2)	0.510

TBV, total blood volume; b.w., body weight; G-CSF, granulocyte colony-stimulating factor; CT, chemotherapy; CE, collection efficiency; TP, throughput. Note: data are presented as median values with minimum–maximum ranges.

**Table 3:** Product characteristics and performance specifications for the Spectra Optia^®^ continuous mononuclear cell collection protocol by type of mobilization regimen.

Variables	Mobilization without Plerixafor		Mobilization with Plerixafor	
	G-CSF (N = 97)	G-CSF + CT (N = 113)	G-CSF (N = 64)	G-CSF + CT (N = 35)
White blood cells (10^9^/L)	216.5 (77.4–438.0)	171.0 (44.0–633.0)	268.0 (117.0–569.0)	185.0 (48.1–460.1)
Platelets (×10^9^/L)	1513 (313–4980)	523 (29–4350)	917 (404–4505)	441 (71–1425)
Hematocrit (%)	1.2 (0.0–5.3)	1.4 (0.0–4.3)	1.1 (0.0–4.2)	1.1 (0.0–3.5)
CD34+ cells total in bag (10^6^)	349 (20–1200)	570 (90–2790)	300 (30–1200)	200 (50–5700)
CD34+ cells/μL processed blood	664 (27–2496)	1165 (168–8173)	565 (39–1936)	379 (94–1241)
CD34+ cells collected (10^6^/kg b.w.)	4.1 (0.2–13.2)	7.3 (1.1–34.9)	3.7 (0.5–11.3)	2.3 (0.7–8.0)
Whole blood processed (L)	19.9 (7.2–29.4)	14.9 (6.4–25.8)	19.5 (11.5–28.5)	18.8 (10.8–29.0)
TBV processes (×times)	4.0 (1.4–5.5)	3.2 (1.5–5.7)	4.0 (2.0–5.0)	4.0 (2.7–5.3)
Apheresis duration (minutes)	346 (155–491)	270 (140–510)	334 (186–480)	329 (220–453)
CD34+ CE2 (%)	52.3 (30.1–119.1)	49.1 (15.0–97.0)	54.0 (26.7–178.0)	52.1 (27.5–115.3)
CD34+ CE1 (%)	69.6 (32.9–154.4)	65.6 (14.2–147.4)	72.9 (39.9–151.6)	67.9 (34.6–165.1)
Platelet loss (%)	42.2 (15.2–60.8)	29.0 (0.0–59.7)	36.5 (3.9–61.3)	29.5 (7.8–50.2)
Platelet CE1 (%)	15.9 (10.4–41.9)	12.8 (2.9–57.6)	15.6 (10.0–70.2)	11.2 (5.6–20.0)
CD34+ TP2 (×10^4^)	3.2 (1.8–7.1)	2.9 (0.8–6.1)	3.4 (1.7–9.5)	3.1 (1.5–7.1)
CD34+ TP1 (×10^4^)	4.3 (1.8–9.2)	3.6 (0.8–8.9)	4.2 (2.4–9.5)	3.8 (1.8–10.2)
CD34+ recruitment factor	2.6 (1.4–5.5)	2.1 (1.1–4.5)	2.7 (1.3–9.3)	2.5 (1.6–5.0)

G-CSF, granulocyte colony-stimulating factor; CT, chemotherapy; N, number of collections; b.w., body weight; TBV, total blood volume; CE, collection efficiency; TP, throughput. Note: data are presented as median values with minimum–maximum ranges.

## Data Availability

Not applicable.

## Supplementary Materials

The following supporting information can be downloaded at: https://www.mdpi.com/article/10.3390/cancers14246259/s1, Figure S1: Relationship (linear regression analysis) between pre-apheresis CD34+ cell count and (a) CE1 or (b) CE2; Figure S2: Relationship (linear regression analysis) between pre-apheresis platelet count and platelet loss (a) overall and (b) by mobilization regimen; Table S1: *p*-values from the Kruskal–Wallis test for between-subgroup comparisons of cell counts, product characteristics, and performance of the collection protocol; Table S2: Summary of simple regression analysis calculating the impact of mobilization method (with or without plerixafor) on CE1 and CE2.
