# Supplementary material for: Impact of Mobilization Strategies on Peripheral Blood Stem Cell Collection Efficiency and Product Quality: A Retrospective Single-Center Study
Source: Cancers (Basel). 2022 Dec 19;14(24):6259. doi: 10.3390/cancers14246259 (PMC9777066; doi:10.3390/cancers14246259)
Supplement: Supplementary file 1 [file cancers-14-06259-s001.zip › cancers-1973794-supplementary.pdf]

cancers

MDPI

Supplementary material

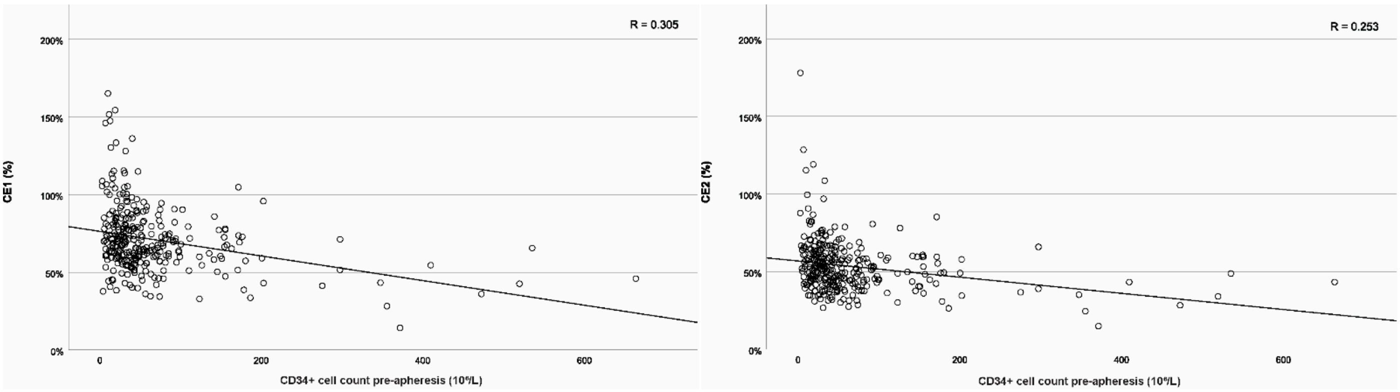

(a) (b)

Figure S1. Relationship (linear regression analysis) between CD34+ cell count pre-apheresis and (a) CE1 or (b) CE2. CE, collection efficiency.

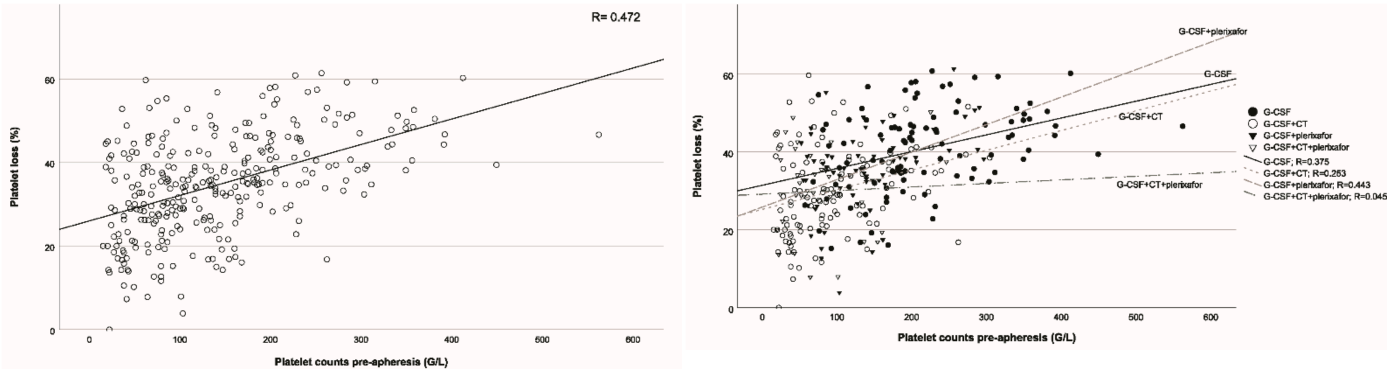

(a) (b)

Figure S2. Relationship (linear regression analysis) between platelet count pre-apheresis and platelet loss (a) overall and (b) by mobilization regimen. G-CSF, granulocyte colony-stimulating factor; CT, chemotherapy.

Table S1. P-values from the Kruskal-Wallis test for between-subgroup comparisons of cell counts, product characteristics and performance of the collection protocol

	G-CSF vs G-CSF + CT	G-CSF vs G-CSF + plerixafor	G-CSF vs G-CSF +CT + plerixafor	G-CSF + CT vs G-CSF + plerixafor	G-CSF + CT vs G-CSF + CT + plerixafor	G-CSF + CT + plerixafor vs G-CSF + plerixafor
Recipient TBV (mL)	0.383	0.890	0.154	0.529	0.407	0.218
Recipient weight (kg)	0.015	0.340	0.034	0.242	0.672	0.208
Pre-apheresis cell counts						
White blood cells (10^9/L)	<0.001	0.750	0.036	<0.001	0.007	0.027
Platelets (10^9/L)	<0.001	<0.001	<0.001	<0.001	0.397	<0.001
Hematocrit (%)	<0.001	0.028	<0.001	<0.001	0.087	<0.001
CD34+ cells (/µL)	<0.001	0.107	0.007	<0.001	<0.001	0.188
CD34+ (%)	<0.001	0.057	0.130	<0.001	<0.001	0.967
Post-apheresis						
CD34+ change (%)	0.006	0.346	0.002	0.145	0.253	0.033
Product characteristics						
White blood cells (10^9/L)	<0.001	0.069	0.013	<0.001	0.993	<0.001

Platelets (10^9/L)	<0.001	<0.001	<0.001	<0.001	0.135	<0.001
Hematocrit (%)	0.264	0.304	0.662	0.040	0.219	0.720
CD34+ x10^6 product	<0.001	0.121	0.003	<0.001	<0.001	0.117
CD34+ cells (10^6/kg b.w.)	<0.001	0.140	0.002	<0.001	<0.001	0.087
Whole blood processed	<0.001	0.981	0.500	<0.001	0.002	0.515
TBV processed	<0.001	0.966	0.698	<0.001	<0.001	0.692
Apheresis time	<0.001	0.911	0.967	<0.001	<0.001	0.901
CD34+ CE 2 (%)	0.056	0.480	0.757	0.016	0.292	0.406
CD34+ CE1 (%)	0.009	0.691	0.125	0.006	0.751	0.081
Platelet loss (%)	<0.001	0.017	<0.001	0.001	0.985	0.014
Platelet CE1 (%)	<0.001	0.289	<0.001	<0.001	0.067	<0.001
CD34+ TP2	0.019	0.374	0.418	0.003	0.395	0.149
CD34+ TP1	<0.001	<0.001	0.023	<0.001	0.737	0.027
CD34+ recruitment factor	<0.001	0.295	0.286	<0.001	<0.001	0.844

G-CSF, granulocyte colony-stimulating factor; CT, chemotherapy; b.w., body weight; TBV, total blood volume; CE, collection efficiency; TP, throughput.

Table S2. Summary of simple regression analysis calculating the impact of mobilization method (with or without plerixafor) on CE1 and CE2

**Model summary**[a]	**R**	**R^2**	**Adjusted R^2**	**SE of the estimate**	
CE1	0.071	0.05	0.002	21.35715%	
CE2	0.114	0.013	0.010	16.77803%	
**ANOVA**[b]	**Sum of squares**	**Degrees of freedom**	**Mean square**	**F-ratio**	**Significance level**[a]
CE1					
Regression	712.611	1	712.611	1.562	0.212
Residual	140031.251	307	456.128		
Total	140743.862	308			
CE2					
Regression	1134.379	1	1134.379	4.030	0.046
Residual	86421.226	307	281.502		
Total	87555.605	308			
**Coefficients**[b]	**Unstandardized beta (*B*)**	**SE *B***	**Standardized beta ß**	***t* test**	**Significance level**
CE1					
(Constant)	70.165	1.474		47.608	0.000
Mobilization	3.254	2.604	0.071	1.250	0.212
CE2					
(Constant)	52.205	1.158		45.090	0.000
Mobilization	4.106	2.045	0.114	2.007	0.046

CE, collection efficiency; SE, standard error.

Notes: [a] Predictors: (constant), mobilization method (0–1 variable; with or without plerixafor)

[b] Dependent variable: CE (%)
